# Hospitals and the War

**Published:** 1914-10-24

**Authors:** 


### HOSPITALS AND THE WAR.

**(From our Special Correspondents.)**

### BATH: THE MEDICAL MAYOR'S EFFORTS.

THE energies of the Mayor of Bath, Dr. Preston King, have been directed during the past week towards the reception of a number of Belgian refugees, for whom, in response to an urgent request, Dr. King undertook, on behalf of the city, to be responsible. Suitable premises were secured, and his Worship's appeal for equipment for the house—sufficient furniture, bedding, etc., was responded to by the local residents within thirty-six hours; and some fifty refugees arrived on Oct. 16. An endeavour is being made to raise the necessary funds to purchase a motor ambulance for the Front. At the moment of writing, the amount received stands at £175. A request was sent to Bath asking whether the city would receive fifty Belgian wounded, and, a reply being forwarded in the affirmative, preparations were made for their reception at the Royal United Hospital and a temporary hospital at Kingswood School, Lansdown. A telegram, however, has now been received stating that all the wounded Belgians have now been sent to Government hospitals.

### BIRMINGHAM: WOUNDED FROM MALINES.

NINETY-FIVE wounded Belgian soldiers arrived in Birmingham late on Thursday afternoon last week, and are now under treatment at the General and Queen's Hospitals and at the Workhouse Infirmary. In spite of their wounds and the terrible privation they suffered during the defence of Malines, Liége, and Louvain, they seemed quite cheerful. Mainly they were infantry of the line, few seriously wounded, and by far the majority suffering from injuries inflicted by artillery fire—shrapnel gashes, concussion, and flash blindness. Some had seen the fighting around Antwerp. The condition of the men was evidence of the rough times through which they had passed. Many of them had lain in the trenches and been covered with filth, while the odd garments that made up some of their outfits suggested the difficulties to which they had been put to clothe themselves. Some were in complete civilian attire; others had perhaps only one article of military uniform.

The soldiers came over from Ostend to Folkestone and travelled by special train to Birmingham, arriving at New Street at 5.20 P.M., and were received by the Lord Mayor. A tremendous crowd had gathered, and the Birmingham public gave to them a welcome as enthusiastic as for our own injured "Tommies." Both at the General and Queen's Hospitals wards had been set apart for their reception. A number of the nurses speak both French and Flemish, so the work of admitting and encouraging was much facilitated.

### BRISTOL.

A FURTHER contingent of wounded, consisting of some 214 Belgians—forty-five of which were stretcher cases—arrived at Bristol on Friday last week (October 16). Dr. Griffiths was in charge of the Red Cross arrangements for their reception, with Dr. Hayman, of the St. John Ambulance Association, and there were in attendance at the railway station the Nos. 7 and 3 Men's Voluntary Aid Detachments and No. 2 Women's Voluntary Aid Detachment, with a contingent of the St. John Ambulance Brigade. After receiving refreshments the patients were speedily removed to hospital in motor ambulances and other motor vehicles, each Belgian wounded soldier being presented with a packet of cigarettes. The patients were chiefly inhabitants of Bruges and Antwerp.

The transport section of the Bristol Red Cross Society appears to be doing good work, for at a recent meeting of the local branch it was stated that in all they had received over 800 cases for transport, the work involving not merely the removal of the wounded from the trains to hospitals, but also numerous transfers of patients from one place to another, and it was seldom a day passed without a request for their services being made.

The Secretary of the Bristol General Hospital announces that the maternity ward, opened on July 27, is now in full working order, and the wives of sailors and soldiers on active service are being received. In view of the large number of refugees, the hospital is prepared as far as it is possible to receive Belgian women into the maternity ward.

### 1,000-BED HOSPITAL AT CAMBRIDGE.

The 1st Eastern General Hospital, which is being erected on the pavilion or block plan on Clare College cricket ground, was originally intended to provide adequate accommodation for 500 patients. Its beds are now to be doubled. The designing, planning, and erection of this hospital reflect great credit upon Colonel J. Griffiths, M.D., M.C., F.R.C.S., and Mr. Skipper, F.R.I.B.A., the architect. A well-known architect and engineer who recently had the opportunity of visiting the institution was heard to remark that he had never before seen a piece of work of such magnitude carried out so expeditiously.

Early on Thursday last week the authorities at the 1st Eastern General Hospital received information that more wounded in all probability would arrive between 1 and 2 o'clock. About 1.45 a Great Western hospital train steamed into the Great Northern portion of Cambridge station bringing from Dover about 125 soldiers of the Belgian Field Army who had received their wounds in the defence of Antwerp. To those familiar with hospitals and hospital construction and management, or who have read The Hospital's recent article on the hospital trains, the admirable and efficient manner in which the train had been equipped as a travelling general hospital in every sense was obvious. About 100 of these wounded Belgians, half of whom were stretcher cases, were speedily conveyed to the 1st Eastern General Hospital, where preparation in the minutest detail had been made for their reception. The remaining twenty-five continued their journey to Wisbech, where preparations had been made for their reception. The gallant Belgians, many of whom appeared to be suffering not only from the result of their wounds but from exhaustion after their terrible experiences, had a most enthusiastic reception.

Members of Cambs. No. 7 Voluntary Aid Detachment, under the command of Dr. Alex Wood, and stretcher bearers from Swavesey, under Commandant E. Frohock, removed the wounded from the train to the motor ambulances and cars lent by Messrs. King and Harper, Messrs. Hallock and Bond, Messrs. Eaden, Lilley and Co., Messrs. J. A. Sturton, Ltd., and Messrs. Laurie and McConnal.

Like the other wounded soldiers who have arrived in Cambridge, the wounded Belgians have been subject to the *x*-rays in the newly fitted up special electrical *x*-ray department at Addenbrooke's Hospital, under Dr. Shillington Scales.

### BELGIANS' STORIES AT CHATHAM.

Many Belgian wounded have arrived at Chatham. A local newspaper representative, visiting at the Drill Hall Military Hospital on Thursday last week, was invited to chat with the Belgian soldiers who have been removed there to be nursed. Several of the patients are well enough to be out in the grounds, and they enjoy the attempts of passers-by to speak Flemish or French, and also their own efforts at English conversation. Small parties of boys gather round the fence and, pointing to various objects, endeavour (with a decided "Chatham" accent) to teach the Belgians some of the more common English words. It is the French of "Stratford-atte-Bowe" over which Chaucer made merry. Other passers-by bring out a few words from a scanty store of half-forgotten French, and cry to their unfortunate friends, "Comment ça va?" Back goes the answer, full of cheeriness, "Bien, merci." Then the exchange of international compliment, an exchange which is being used more and more as the number of refugees on British soil increases, "Vive la Belgique!" "Vive l'Angleterre!"

Speaking in French, one of the 106 Belgians, many of whom are suffering from pleurisy, pneumonia, rheumatism, and like complaints, said: "Belgium is ruined, devastated, burnt to ashes. Our fields are blackened, our homes destroyed, our loved ones murdered, taken prisoners, or exiled. We have had to stand by, knowing that all we have striven for in the past has been ruthlessly destroyed, and that for years, perhaps decades, Belgium can be nothing more than it is now—a vast desert. And everything is the work of the Bosches." Only those who heard him can imagine the infinite amount of hatred and contempt he poured into the word "Bosches." "Bosche," to the French and Belgians, has come to mean all that is evil; and who can wonder?

This warrior, who pointed to a scar he had received from a bayonet thrust in the wrist, had been engaged in six battles, and was full of admiration for the way the Germans fight with their big guns. "They fire," he said, "from behind elaborately arranged shelters; it is often difficult to locate them, and their fire is terrible. But when we get up close with them—then it is our game. If we just show them a bayonet they run, run, run—like rabbits. Bayonets are cold, steely things; they cut deep. When are we going back? We can't tell. We have no homes to go to; no cheery hearths; no friends to welcome us. Some have fled to Holland; but we don't know where they are."

Forty-one more patients were admitted to the Strood Voluntary Hospital on Thursday last week. Many of the men's clothes were in a deplorable state.

### COLCHESTER.

Arrangements are now practically completed at the Essex County Hospital for the reception of sick and wounded from the Expeditionary Force. An appeal was recently made for an additional twenty bedsteads, and a generous response was received within a few days, so that the needed additions will be shortly available. Although no wounded have yet been drafted to the wards, a number from Territorial regiments in the county have been treated in both the medical and surgical wards, and as the military hospital has recently received several drafts it is believed that the authorities will shortly be utilising all the accommodation now available. Dr. Sydney W. Curl, hon. physician, has been called up for service and is now stationed at the base hospital at Cambridge.

### DONCASTER: THE MANSION HOUSE HOSPITAL.

UNDER the charge of Commandant Mrs. Pickering and Miss Smith, the temporary war hospital in the Doncaster Mansion House is supplied with everything but patients at present. Already a number of wounded soldiers have been drafted to Lady Galway's war hospital at Serlby, only a few miles south of Doncaster. In the banqueting room of the Mansion House fifty beds are in readiness for their occupants, and a neighbouring room has been partially fitted up for minor operations. The Doncaster Voluntary Aid Detachment was strong in nurses before the war broke out, and since August the work of qualifying young women for Red Cross usefulness has proceeded with enthusiasm. Fifty can be drafted in at short notice, and of these about thirty are fully trained. Emergency lectures in nursing and first-aid are being given, and about seventy members of the Detachment have passed the examination. By the willing co-operation of the Doncaster Royal Infirmary authorities all the V.A.D. nurses are receiving a course of practical training in that institution, attending in batches from 2 to 7 o'clock each day, and being initiated into general ward work and the duty of the operating theatre. This co-operation is the more readily afforded inasmuch as the infirmary governors are precluded, by extreme pressure on an inadequate building, from offering any accommodation to wounded fighting men.

Of the male section of the St. John Ambulance Association in the town—almost exclusively recruited from the ranks of the local railwaymen—the members have volunteered practically *en bloc* for duty in the R.A.M.C., and weekly drills and first-aid practices are now being held.

### GRAVESEND: THE OVERFLOW FROM DOVER.

NEARLY fifty wounded Belgian soldiers and French Marines were admitted on Wednesday last week into the Gravesend General Hospital, and now lie side by side with the English "Tommies," who, as may be imagined, gave them a hearty welcome as they were taken into the wards. Sick and fatigued, crippled in arms and legs, limping, hopping with the assistance of a friendly arm, or with the aid of sticks and crutches, thus they came to seek our hospitality and treatment. Representatives of many regiments—infantry of the line, artillery, lancers, etc., and French Marines—all kinds and conditions of uniforms, torn and bearing evidence of hard and terrible experiences undergone. Many without uniforms, clad in anything they could get, without boots, in stockinged feet or felt slippers, all completely tired out and looking worn and dejected. They were put quickly into bed and given food and hot drinks, and their expressions of gratitude were unending.

So great was the congestion of wounded at Dover and Folkestone that towards midnight the hospital authorities were informed that another batch of fifty had been sent for care and treatment, but as all the beds were full—nearly one hundred—the Gravesend and Northfleet Voluntary Aid Detachment stepped into the breach, and in a very short time got ready the fifty beds in the temporary hospital in the old Yacht Club House. Their effort was very praiseworthy, and their ready and successful response to such an urgent appeal could have only been the result of much forethought, hard work, and capable organisation. The patients, although expected much earlier, did not arrive until about 4.30 on Thursday morning, but, notwithstanding the early hour, quite a number of people assembled at the station to give them a welcome. Transport arrangements for both convoys were expeditiously carried out by owners of motor-cars assisted by the Voluntary Aid Detachment stretcher squad.

### HASTINGS: INFLUX OF WOUNDED AND REFUGEES.

DURING the last week 116 wounded Belgians arrived in Hastings, forty-one of whom were admitted into the East Sussex Hospital, twenty-four, including three officers, into the Buchanan Hospital, and sixteen into a temporary hospital in the Holmesdale Gardens, which has been fully equipped and recently opened by the St. John Voluntary Aid Detachment, while thirty-five were taken in at the Convent of Our Lady at Filsham Park, St. Leonards. The men are not seriously wounded, there being chiefly bullet wounds in the arms and legs, and will soon be able to leave. In addition to the wounded soldiers a further influx of Belgian refugees arrived in Hastings on Wednesday and Thursday last week.

### LEICESTER: GENERAL FORD'S VISIT.

THE event of the past week was the visit of Surgeon-General Ford, Deputy Director Medical Services, Northern Division (York command). General Ford made a thorough inspection of the 5th Northern General Hospital and congratulated the officer in command, Lieut.-Col. Harrison, and his staff on the efficiency of the organisation. About 250 patients now remain, some seventy having been discharged during the week. The entertainments most kindly given by local artistes two evenings every week continue to give great pleasure. A most generous offer has been made to the County Director Voluntary Aid Detachments, Mr. A. W. Faire, by the Brewers and Licensed Trade of Leicester, through Messrs. T. W. Everard, J.P., and Walter G. Turner, to provide the sum of £200 for ambulance transport in connection with the Red Cross Service. This offer has been gratefully accepted, and Mr. Faire will now have at his disposal the means of transit which has been for some time felt to be needed.

### A Clearing Hospital for Foreign Service.

Orders have been received for the mobilisation of the North Midland Divisional Clearing Hospital, R.A.M.C. Lieut.-Col. W. Pemberton Peake is the commanding officer, and it is expected that the unit will shortly be dispatched on foreign service. Its *personnel* consists of eight officers and seventy-seven non-commissioned officers and men, whose duty it is to connect the field hospitals behind the firing-line with the base hospitals and ports of embarkation. The efficiency of this clearing hospital means much to the sick and wounded, as it is generally recognised that, if wounded men can be received into the military hospitals of England within the first three or four days of casualty, the risks of infection are very considerably reduced.

### NORWICH: TENTS FOR THE WOUNDED.

One hundred wounded, ninety-nine British and one Belgian, from Lille arrived at the Norfolk and Norwich Hospital at midnight on Friday last week. They were conveyed from Dover in the special hospital train and were met at Norwich Thorpe Railway Station by members of the board of management and the local Red Cross Society, the latter rendering very valuable services in assisting the patients from the train to the conveyances in readiness. They were received at the hospital by the medical and nursing staffs in less than one hour. Early last Sunday morning a further telegram was received informing the hospital authorities that another contingent of 110 wounded British soldiers would arrive from Southampton during the afternoon. Arrangements were at once made for transferring some of the patients suffering from slight ailments to various convalescent homes in the county, and about thirty were dispatched in motor-cars about midday.

The new contingent arrived at Norwich about 6 p.m., and were conveyed to the hospital in a similar way as on the Friday evening. The tents specially erected for the purpose are now all in use, as well as three wards in the hospital and the King Edward block. The cases that arrived on Sunday evening were more serious than the first contingent, as many as thirty being "cot" cases. The arrangements for the reception of the patients at the hospital were carried out admirably under the superintendence of Miss Cann, the matron, and Mr. F. G. Hazell, the secretary.

Mr. Charles Larking, the chairman of the board of management, Mr. R. E. Horsfall, chairman of the house committee, Mr. R. Laurence, Canon Fitzgerald, and other members of the board of management were present to receive the patients.

### ROCHESTER.

About fifty wounded from the recent fighting in France were received at St. Bartholomew's Hospital, Rochester, on Thursday last week. They were men from the 1st Gordons, 1st Duke of Cornwall's Light Infantry, 1st Dorset, 2nd Manchester, 13th East Surrey, 1st Middlesex, 1st Bedfordshire, 2nd King's Own Scottish Borderers, 1st Northampton Fusiliers, Royal Field Artillery, 3rd Worcestershire, and 1st Cheshire. One of the patients remarked that it was a great treat to find himself in a bed and to have clean clothing. He had not changed his shirt since August 15.

### SUNDERLAND: THE FIRST CONVOY.

Sunderland received its first convoy of wounded soldiers from the Front on Saturday last. Numbering 100 sick and wounded, including twenty-five cot cases, the party left Southampton at 9 a.m. and reached Sunderland about 6.30 p.m. Here they met with a splendid reception on the route from the Central Station to the Royal Infirmary.

The arrangements for the conveyance of the men to the infirmary were in the hands of Mr. Claude B. Palmer, County Director Northumberland and Durham St. John Voluntary Aid Detachment, assisted by Mr. A. Seymour Wood, Inspector of Stores, St. John Voluntary Aid Detachment, and were expeditiously carried out, the slightly injured being conveyed in motor-cars lent by private owners, and the serious cases in horse ambulances from the various collieries in the district. On arrival at the infirmary, the wounded men were received by the Mayor, Alderman S. Richardson, J.P., and members of the house committee, while the whole of the honorary and resident staff was present to attend to them.

### SWANSEA: ACTIVE SERVICE LIST.

The committee of the Swansea General Hospital placed ninety beds at the disposal of the War Office, which offer has been accepted.

The honorary medical staff and the nursing staff is much depleted through the loss of many officers and nurses through absence on service. In addition to Dr. Lancaster, Mr. W. F. Brook, Dr. Elsworth, Dr. Reid, and Dr. F. G. Thomas, who are attached to the Cardiff Base Hospital, Dr. Daniel E. Evans, hon. assistant physician, Mr. Alban Evans, hon. surgeon to the throat, nose, and ear department, Dr. H. E. Quick, hon. assistant ophthalmic surgeon, and Dr. Chiles Evans are serving with different regiments throughout the country; whilst Mr. C. Leonard Isaac, hon. surgeon, is the senior medical officer of the home defences in Swansea.

Then as to the nurses. Miss M. P. Scovell (who has done excellent work since she has held the office of matron at the Swansea Hospital) is at the 4th London General Hospital, King's College Hospital, at which latter place she was trained, and with her are two of her sisters from Swansea, Miss Gent, the night superintendent, and Miss Catt, of the Penllergaer male medical ward; Miss Gammie, sister of the Talbot male surgical ward, is at the Newcastle Military Hospital; Miss Dunbar, the *x*-ray sister, is at the Front; and Miss Harbutt, theatre sister, and Miss Sandbrook, the casualty and out-patient sister, are at the 3rd Western General Hospital, Cardiff. Besides these, Miss Black, staff nurse, is stationed at Aldershot.

**[Owing to the exceptional pressure on our space sever other accounts have been unavoidably held over.]**

